# *APOE* Genotyping in Cognitive Disorders: Preliminary Observations from the Greek Population

**DOI:** 10.3390/ijms26157410

**Published:** 2025-08-01

**Authors:** Athanasia Athanasaki, Ioanna Tsantzali, Christos Kroupis, Aikaterini Theodorou, Fotini Boufidou, Vasilios C. Constantinides, John S. Tzartos, Socrates J. Tzartos, Georgios Velonakis, Christina Zompola, Amalia Michalopoulou, Panagiotis G. Paraskevas, Anastasios Bonakis, Sotirios Giannopoulos, Paraskevi Moutsatsou, Georgios Tsivgoulis, Elisabeth Kapaki, George P. Paraskevas

**Affiliations:** 1 2nd Department of Neurology, School of Medicine, National and Kapodistrian University of Athens, “Attikon” General University Hospital, 12462 Athens, Greece; athanasia.athan@yahoo.gr (A.A.); docjo1989@gmail.com (I.T.); katetheo24@gmail.com (A.T.); jtzartos@gmail.com (J.S.T.); chriszompola@yahoo.gr (C.Z.); aliam2204@gmail.com (A.M.); panprskvs7@gmail.com (P.G.P.); bonakistasos@med.uoa.gr (A.B.); sgiannop@uoi.gr (S.G.); tsivgoulisgiorg@yahoo.gr (G.T.); 2 Department of Clinical Biochemistry, School of Medicine, National and Kapodistrian University of Athens, “Attikon” General University Hospital, 12462 Athens, Greece; ckroupis@med.uoa.gr (C.K.); pmoutsatsou@med.uoa.gr (P.M.); 3 1st Department of Neurology, School of Medicine, National and Kapodistrian University of Athens, Neurochemistry and Biological Markers Unit, “Eginition” Hospital, 11528 Athens, Greece; fboufidou@med.uoa.gr (F.B.); vassilis.kon@hotmail.com (V.C.C.); ekapaki@med.uoa.gr (E.K.); 4 Tzartos Neuro Diagnostics, 11523 Athens, Greece; stzartos@gmail.com; 5 2nd Department of Radiology, National and Kapodistrian University of Athens, Research Unit of Radiology, “Attikon” General University Hospital, 12462 Athens, Greece; giorvelonakis@gmail.com; 6 Department of Neurology, University of Tennessee Health Science Center, Memphis, TN 38163, USA

**Keywords:** Alzheimer’s disease, dementia, cognitive impairment, apolipoprotein E, amyloid beta, tau protein, phospho-tau protein

## Abstract

Alzheimer’s disease (AD) is the most common cause of cognitive decline. Among the various susceptibility genes, the gene of apolipoprotein E (*APOE*) is probably the most important. It may be present in three allelic forms, termed ε2, ε3 and ε4, and the most common genotype is the ε3/ε3. Recently, it has been observed that subjects with the ε4/ε4 genotype may show near-full penetrance of AD biology (pathology and biomarkers), leading to the suggestion that ε4 homozygosity may represent a distinct genetic type of AD. The aim of the present study was to investigate the role of ε4 homozygosity or heterozygosity in the presence or absence of the AD biomarker profile in patients with cognitive disorders in the Greek population. A total of 274 patients were included in the study. They underwent *APOE* genotyping and cerebrospinal fluid (CSF) biomarker profiling. The presence of ε4 was associated with a lower age of symptom onset and decreased amyloid biomarkers (irrespective to AD or non-AD profiles), and predicted the presence of an AD profile by a positive predictive value approaching 100%. In conclusion, the ε4 allele has a significant effect on the risk and clinical parameters of cognitive impairment and AD in the Greek population, while the ε4/ε4 genotype may be highly indicative of the (co)existence of AD in cognitively impaired patients.

## 1. Introduction

Alzheimer’s disease (AD) is the most common cause of cognitive decline, usually presenting as a hippocampal amnestic disorder [1]. Atypical clinical phenotypes may occur, including logopenic or other language presentations, posterior cortical atrophy, corticobasal syndrome and frontal presentations, whilst cases mixed with cerebrovascular disease or Lewy body pathology are not uncommon [2,3]. Pathologic hallmarks include extracellular amyloid deposition and intraneuronal deposition of hyperphosphorylated tau protein, as well as microglia activation, neuroinflammation and synaptic and neuronal loss [4]. Since the amyloid and tau pathological/biochemical processes are reflected in the cerebrospinal fluid (CSF) or can be assessed by positron emission tomography (PET), the use of such biomarkers has been incorporated in recent research or diagnostic criteria and classifications [5,6,7,8]. In the CSF, Alzheimer’s disease is characterized by reduced levels of amyloid beta (Aβ) with 42 amino acids (Aβ_42_), whilst controlling Aβ_42_ for the levels of Aβ with 40 amino acids (Aβ_40_) in the form of the Aβ_42_/Aβ_40_ ratio may better reflect the amyloid process. Concomitantly, the levels of phosphorylated tau protein, such as tau phosphorylated at threonine 181 (τ_P181_), are increased. Thus, amyloid positivity (A^+^), defined as reduced CSF levels of Aβ_42_, or, preferably, reduced Aβ_42_/Aβ_40_ and tau positivity (T^+^), defined as increased levels of τ_P181_, formulate the CSF profile of AD, typified as A^+^T^+^ [5] or A^+^T_1_^+^ (since τ_P181_ increases very early in the AD process, as a result of amyloidogenesis) [6]. In this context, AD may be viewed as a biological [6] or clinical–biological entity [7], with the presence (or absence) of AD being diagnosed according to the abovementioned biomarker profile, irrespective of the clinical phenotype (typical or atypical) and the severity of symptoms (mild cognitive impairment or dementia). Although it has been proposed that A^+^ may suffice for the diagnosis of AD [6], many authorities suggest relying on the A^+^T^+^ profile and, in the case of A^+^T^−^ or A^−^T^+^ profiles, using other alternative methods, such as amyloid PET [3,9]. Another alternative method may be the use of the hybrid τ_P181_/Aβ_42_ ratio [6].

Among the various susceptibility genes, the gene of apolipoprotein E (*APOE*) at chromosome 19 is probably the most important [10]. It may be present in three allelic forms, termed ε2, ε3 and ε4, and the most common genotype in the general population (including the Greek population) is ε3/ε3 [11], which is accompanied by a lifetime risk for developing AD of ~10% [12]. The presence of the ε4 allele is considered a major genetic risk factor for the development of AD, adversely affecting Aβ production, fibrilization, accumulation and clearance; tau hyperphosphorylation, aggregation and spread; neuroinflammation; and the function of neural networks [4,10]. Vascular mechanisms, including cerebral amyloid angiopathy (CAA), may also be involved [13]. This action of ε4 occurs in a “dose-dependent” manner, and it has been estimated that ε4 heterozygosity increases the lifetime risk for AD to ~30% and ε4 homozygosity increases it to ~65%, concomitantly decreasing the age of symptom onset [12]. On the other hand, ε2 has a protective role and decreases the risk of AD [14]. However, accumulating evidence suggests that the ε4/ε2 genotype may be similar to ε4/ε3, showing increased neurodegeneration, yet with a relatively slower rate of progression [15].

Recently, it has been observed that subjects with the ε4/ε4 genotype may show near-full penetrance of AD biology (pathology and biomarkers) with an approximate age of symptom onset of 65 years, leading to the suggestion that ε4 homozygosity may represent a distinct genetic type of AD (somehow similar to autosomal-dominant AD or Down syndrome) [16]. Then, the question arises as to whether ε4/ε4 has a more “deterministic” role, in contrast to ε4 heterozygosity, which is a risk factor. The aim of the present study was to investigate the role of ε4 homozygosity or heterozygosity in the presence or absence of the AD CSF biomarker profile in patients with cognitive disorders in the Greek population. 

## 2. Results

A total of 274 participants presenting with cognitive disorders were included in the study, and their demographic, clinical and CSF biomarker data are presented in Table 1. No difference in gender was observed across ε4 homozygotes, ε4 heterozygotes and non-ε4 carriers (χ^2^ test 1.033, *p* = 0.597), or across ε4 homozygotes, ε4 heterozygotes, and ε3/ε3 and combined ε3/ε2 plus ε2/ε2 groups (χ^2^ test 1.037, *p* = 0.792). No differences were observed in respect to education, Mini Mental State Examination (MMSE) scores and Addenbrooke’s Cognitive Examination—Revised version (ACE-R) scores. Both ε4 homozygotes and ε4 heterozygotes showed significantly higher scores for Entorhinal Cortex Atrophy (ERICA) as compared to non-ε4 carriers, but comparable Medial Temporal Atrophy (MTA) scores.

### 2.1. APOE and Age of Disease (Symptom) Onset

**Figure 1:**
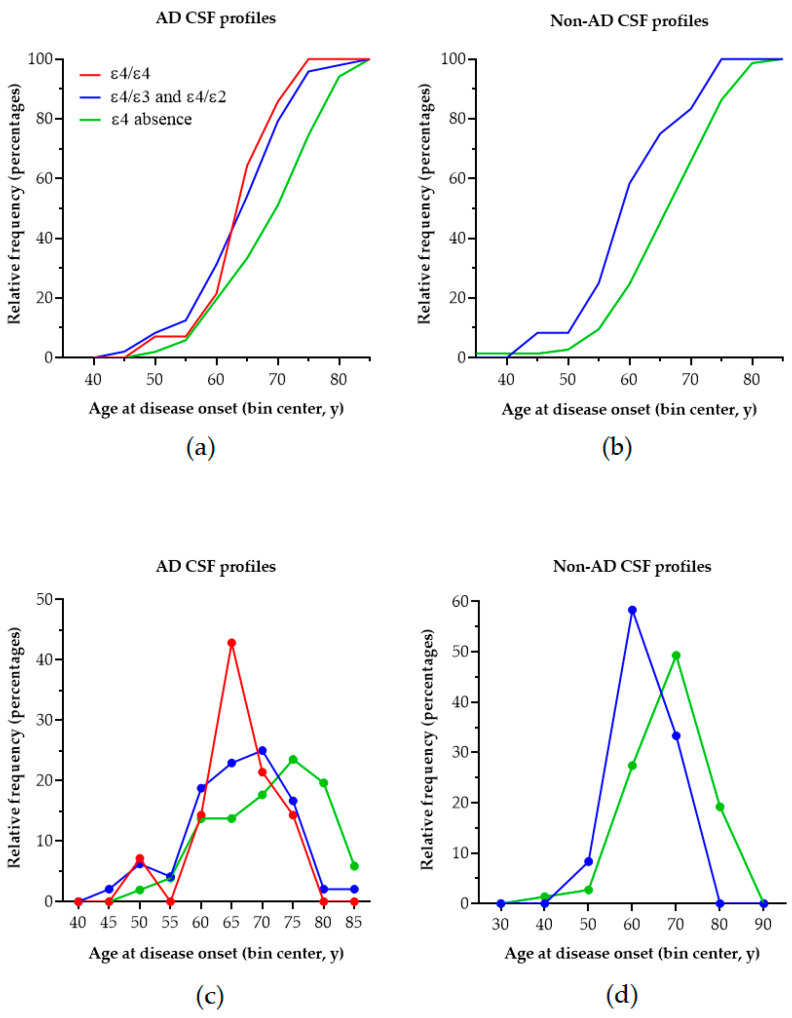
The cumulative frequency distribution of age at disease onset according to the APOE genotype in patients with AD (**a**) and non-AD (**b**) biomarker profiles. Although shifted to the left (earlier age of onset compared to ε4 absence), there is no clear-cut difference between ε4 homozygotes and ε4 heterozygotes with AD. (**c**) The peak incidence of age of onset of AD becomes progressively earlier with an increasing ε4 load in individuals with AD. (**d**) Interestingly, the peak incidence of age of onset occurs earlier with an increasing ε4 load in non-AD patients as well.

Patients with the ε3/ε3 genotype presented with a significantly older age and age of disease onset as compared to ε4 heterozygotes, whilst the latter did not differ significantly compared to ε4 homozygotes. By visual inspection of the cumulative frequency of age at disease onset (Figure 1), it is obvious that, for patients carrying at least one ε4 allele, this is shifted towards earlier ages (~10 years earlier) in patients with AD biomarker profiles, but also in patients with non-AD profiles. In AD, the peak of age of onset in ε4 homozygotes occurs 5 years earlier compared to in ε4 heterozygotes and 10 years earlier compared to in patients with no ε4 allele. In individuals with non-AD profiles, the peak frequency in heterozygotes occurs ~10 years earlier compared to in individuals with the absence of ε4.

### 2.2. APOE and CSF Biomarkers

Differences were noted in respect to CSF biomarker levels. Since assessments were conducted in three different laboratories with different cut-off values, general analysis of covariance models with age as a covariate and laboratory, sex and *APOE* genotype as co-factors was performed for all biomarkers, in order to control for the above parameters. Except for Aβ_40_, all models showed significant results, with lower Aβ_42_ (*p* < 0.001) and Aβ_42_/Aβ_40_ levels (*p* < 0.0001) and higher τ_P181_ (*p* < 0.001), total tau (τ_T_) (*p* = 0.02) and τ_P181_/A_β42_ (*p* < 0.001) levels in patients with at least one ε4 allele vs. patients with the absence of ε4. A significant effect of laboratory was also observed (*p* < 0.0001).

**Figure 2:**
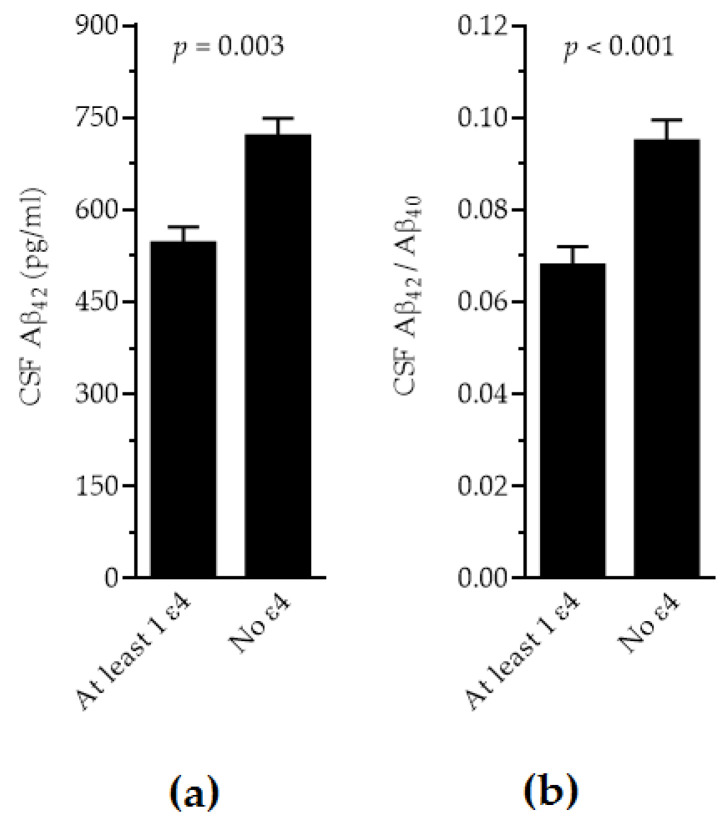
Adjusted levels of CSF Aβ_42_ (**a**) and Aβ_42_/Aβ_40_ (**b**) in individuals with ε4 presence vs. ε4 absence.

When the disease type (AD vs. non-AD profiles) was introduced in the models additionally, all biomarkers showed significant differences by disease type (*p* < 0.0001, except for Aβ_40_ with *p* = 0.04) and by laboratory (*p* < 0.001). For τ_P181_, τ_T_ and the τ_P181_/A_β42_ ratio, the significant differences by *APOE* genotype were lost. However, for Aβ_42_ levels and the Aβ42/Aβ_40_ ratio, significantly lower levels were observed in individuals with the presence of ε4 vs. the absence of ε4 (Figure 2, *p* = 0.003 and *p* < 0.001, respectively). Sex and age did not affect any of the models significantly.

### 2.3. APOE and Biomarker Profiles

Based on the CSF biomarker assessments, CSF profiling of patients was conducted according to the AT(N) classification system [5] (Table 2). For those with the A^+^T^−^ profile, the τ_P181_/A_β42_ ratio was used [6] and, when increased, these patients were considered as having AD and were added to those with the A^+^T^+^ profile. On the other hand, a normal τ_P181_/A_β42_ ratio was considered to be evidence of AD absence, and such patients were classified as non-AD patients. None of the typical non-AD profiles (A^−^T^+^ and A^−^T^−^) showed abnormal levels of the τ_P181_/A_β42_ ratio.

**Figure 3:**
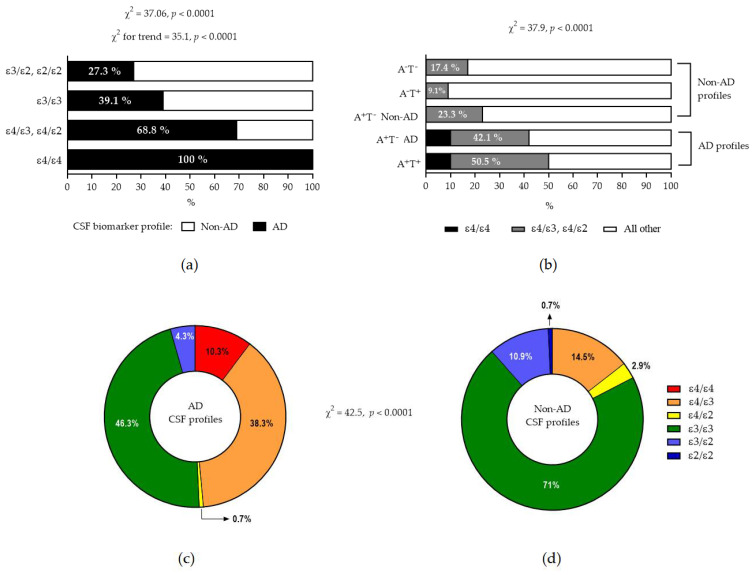
(**a**) Increasing frequency of AD profile from ε4 absence to ε4 presence. Note that all ε4 homozygotes had AD biomarker profiles. (**b**) Increased frequency of ε4 presence in patients with AD biomarkers (the terms A^+^T^−^ AD and A^+^T^−^ Non-AD indicate A^+^T^−^ patients with an increased or normal τ_P181_/A_β42_ ratio, respectively). Note that ε4/ε4 was found only in patients with AD profile. (**c**,**d**) show pie charts of relative frequencies of various APOE genotypes in AD and non-AD biomarker profiles, respectively.

A statistically significant increasing frequency of AD biomarker profiles can be observed when moving from ε3/ε2 and ε2/ε2 to ε3ε3, then to ε4 heterozygosity, and finally to ε4 homozygosity (Figure 3a, *p* < 0.0001). Conversely, the presence of ε4 was significantly more frequent in patients with AD biomarkers as compared to patients with non-AD profiles, and, in addition, ε4 homozygosity was observed only in AD profiles (Figure 3b–d, *p* < 0.0001).

Simple Fisher’s exact tests showed significant results for the prediction of the AD biomarker profile according to the APOE genotype (Table 3). Odds ratios for AD were high, accompanied by relatively low sensitivities, but specificities were high. For ε4 homozygocity, the specificity and positive predictive values approached 1 (100%), although the negative predictive values lower.

In order to confirm the above results by better testing predictive ability and controlling for the possible effects of sex and education, we performed two logistic regression models (first: ε4 presence vs. ε4 absence; second: ε4 homozygosity, ε4 heterozygosity and combined ε3/ε2 and ε2/ε2 vs. the “neutral” ε3/ε3 as a reference) (Table 4). Sex (but not education) affected the models significantly. Again, the odds ratios for ε4 presence were high, especially for ε4 homozygosity, with the results of both the logistic regression and the simple Fisher’s exact tests approaching or exceeding 38.

### 2.4. APOE and Clinical Phenotypes

**Figure 4:**
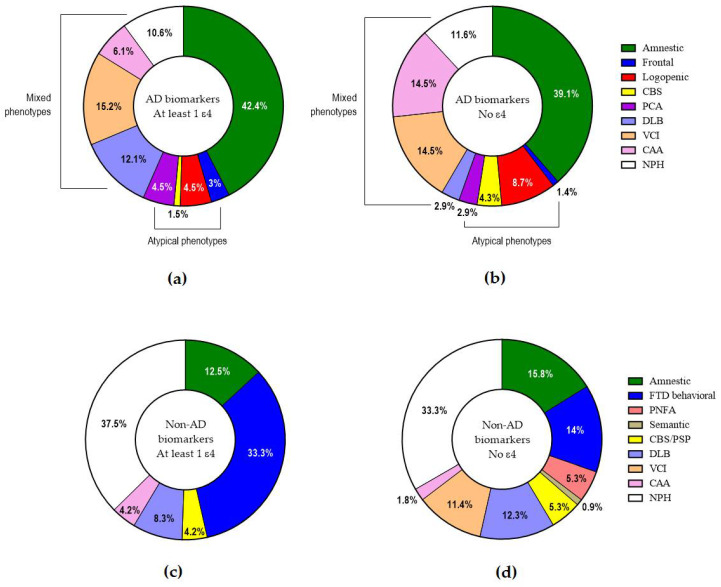
Clinical phenotypes/presentations in AD with at least 1 (**a**) or no ε4 allele (**b**) and in non-AD with at least 1 (**c**) or no ε4 allele (**d**).

The frequencies of the various clinical phenotypes/presentations (Figure 4) did not show any significant differences in respect to the presence or absence of ε4. Within AD, the presence or absence of ε4 did not differ among typical, atypical or mixed presentations.

## 3. Discussion

In this study, the A^+^T^+^ profile was used as a diagnostic tool of AD [5,9,17]. On the other hand, the A^+^T^−^ profile may be heterogeneous, and it has been observed that some AD patients may present with normal or marginal τ_P-181_ levels, especially in the case of atypical or mixed presentations [18,19,20,21]. In such cases, an abnormal level of the hybrid τ_P-181_/Aβ_42_ ratio may be helpful as an indication of AD presence [6,22]. Thus, A^+^T^−^ patients with an increased τ_P-181_/Aβ_42_ ratio were also considered to have AD, and they were added to those with the A^+^T^+^ profile. However, it is recognized that some A^+^T^−^ patients differ in many clinical, neuropsychological and genetic aspects compared to individuals with AD [23,24], and may belong to a different group [25]. Thus, an A^+^T^−^ profile with a normal τ_P-181_/Aβ_42_ ratio may be considered suggestive of amyloid *pathology* (Alzheimer’s *neuropathological change*, Alzheimer’s *continuum*), but not Alzheimer’s *disease* [5], and such patients were added to the other non-AD profiles (A^−^T^+^ and A^−^T^−^).

The main finding of the present study was that all of our cognitively impaired patients with the ε4/ε4 genotype proved to have AD according to their CSF biomarker profile, irrespective of the phenotype, and vice versa, homozygosity was present only in AD patients. The positive predictive value of 100% makes almost certain the (co)existence of AD in an ε4/ε4 person developing cognitive impairment. Although this observation is partly in accordance with the suggestion that ε4 homozygocity may represent a distinct genetic form of AD [16], we must keep in mind that the present study was conducted as a cross-sectional study of already-symptomatic patients, and did not examine how many ε4 homozygotes will develop AD during their lifetime. Furthermore, although the specificity of ε4 homozygocity for AD presence is 100% with a narrow 95% confidence interval, this is not true for the positive predictive value. The latter is 100%, (Table 3), but the relatively wide 95% confidence interval (77–100%) may indicate that AD might not be present in all ε4/ε4 patients. Indeed, it has been shown that ε4 homozygosity becomes “deterministic” only with the coexistence of loss-of-function *SORL1* mutations [7,26].

We also observed that in AD, ε4 homozygosity seemed to have a peak incidence of symptom onset that was 5 and 10 years earlier compared to that for ε4 heterozygosity and ε4 absence. This is similar to other observations [27]. The cumulative incidence of age of symptom onset for ε4 presence seemed to be shifted towards earlier ages compared to that for ε4 absence; however, there was no clear-cut difference between ε4 homozygosity and heterozygosity, and the mean age of onset between these two groups was comparable. This could be attributed to the low number of patients in the ε4/ε4 group and/or to other risk factors present in the studied population. However, this shift towards earlier ages in the presence of ε4 was also observed in non-AD patients, indicating that ε4 may exert an effect in other causes of cognitive impairment. Indeed, ε4 may be a risk factor or modifier for dementia with Lewy bodies (DLB), vascular cognitive impairment (VCI), cerebral amyloid angiopathy (CAA) and, possibly, even for the frontotemporal dementia-amyotrophic lateral sclerosis complex [13,28,29,30,31,32,33,34].

We also observed that ε4 presence is associated with abnormal amyloid and tau biomarkers compared to ε4 absence, and this has been reported previously [10] with the aid of positron emission tomography [35,36]. However, we observed that, when controlling for the type of dementing disease, only amyloid-related markers were adversely affected by ε4, indicating an independent effect of ε4 on brain amyloidogenesis.

There are some limitations to the present study. (a) The number of patients was relatively low, but this is an inherent problem in one-center studies. Subsequently, the limited percentage of ε4 homozygotes, despite it being comparable with that for larger cohorts [16], constrains the generalizability and the statistical power for this subgroup. (b) The results of the present study should be viewed with caution in terms of population characteristics, since all patients were of Greek ancestry, and no data were available for other ethnicities. (c) Despite maximal effort for accurate biomarker-based diagnosis (including participation in external quality control programs), all of our patients are still alive, and no pathological verification (which is the gold standard for AD diagnosis) is available. Furthermore, the presence of some neurodegenerative co-pathologies cannot be excluded. Biomarkers do suggest the presence or absence of AD, but they cannot clarify whether AD is the most important of the co-pathologies which could be present [3]. (d) Polymorphisms/mutations in other genes known to affect the risk of AD [1,4] were not studied, notably SORL1and TREM2, which are of great importance as risk genes. (e) The effects of cardiovascular risk factors [37] such as hypertension, diabetes and dyslipidemia were not investigated. (f) The cross-sectional design of the presented study limits the capacity to identify disease progression over time, underscoring the necessity of longitudinal data. Thus, the present study should be considered as a pilot study, the observations should be considered preliminary and the results should be interpreted with caution. Further studies are needed that include a larger number of participants and take into account other mutations in risk genes and the effect of cardiovascular risk factors.

In conclusion, ε4 homozygosity seems to be strongly related to AD and to predict its presence. Some effects, especially on the age of onset of cognitive impairment and on amyloidogenesis, may be independent of the presence or absence of AD.

## 4. Materials and Methods

### 4.1. Study Design

All patients were consecutively examined in the outpatient clinic and hospitalized in the 2nd Department of Neurology between May 2021 and December 2024. The study was cross-sectional and received the approval of the Ethics Committee and the Scientific Board of “Attikon” Hospital (project identification codes of approval: 157, 16 March 2021 and A13, 7 April 2021, respectively), and it was conducted in accordance with the ethical guidelines of the 1964 Declaration of Helsinki. Written informed consent was provided by all participants and/or their next of kin.

The criterion for inclusion was the presence of a primary cognitive disorder due to a neurodegenerative or vascular brain disorder. Normal-pressure hydrocephalus was also included, since it may coexist and interact with AD or other primary disorders. For diagnosis, internationally accepted criteria/guidelines were used [2,3,5,7,38,39,40,41,42,43,44,45,46]

The criteria for exclusion were as follows: (a) the presence of a major systemic disorder (including malignancies and autoimmune disorders) or major psychiatric disorder (including chronic schizophrenia and mood disorder); (b) the presence of secondary causes of cognitive impairment, such as a central nervous system infection (including neurosyphilis), tumors, subdural hematoma or autoimmune/paraneoplastic encephalopathy; (c) the presence of vitamin B_12_ deficiency or hypothyroidism (allowed if restored for at least 6 months); (d) a history of stroke within the last 6 months; and (e) denial or contraindications for lumbar puncture, including anticoagulation or a low platelet count.

**Figure 5:**
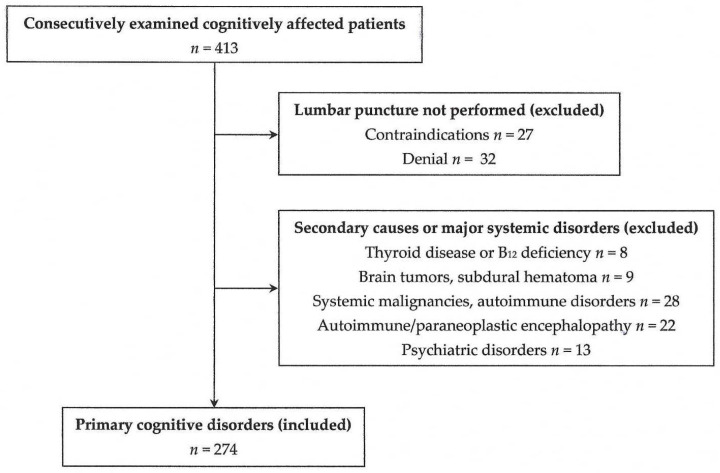
A flow chart of the participants in the present study.

Since this was intended to be a pilot study, data until the end of December 2024 were collected for preliminary observations, without the prior use of power analysis (Figure 5). In addition, no control group was used, since the study aimed to examine the role of the *APOE* genotype within cognitively impaired patients, and not to make comparisons with the general population.

### 4.2. Patient Clinical Approach

Initially, a detailed history and complete physical and neurological examination results, as well as neuropsychological testing results, were recorded for all patients. The Mini Mental State Examination (MMSE) [47] and Addenbrooke’s Cognitive Examination—Revised version (ACE-R) [48], both of which are validated in Greece [49,50], were used for estimating the degree of cognitive decline. Routine 3-Tesla magnetic resonance imaging (MRI) scans with 3D T1W images were performed, and Medial Temporal lobe Atrophy (MTA) [51] and Entorhinal Cortex Atrophy (ERICA) [52] visual scores were used as crude estimates of atrophy of the hippocampal formation and the (trans)entorhinal cortex, respectively.

### 4.3. Lumbar Puncture and CSF Biomarker Assessments 

Following overnight fasting, lumbar puncture was performed at the L4–L5 interspace using a standard, 21–22G, Quincke-type needle, and CSF was collected in polypropylene tubes, handled and stored as previously described [53]. The classical CSF biomarkers, Aβ_42_, Aβ_40_, τ_P-181_ and total tau (τ_T_), were assessed in 3 laboratories in Athens, Greece, according to locality.

Laboratory 1 (Neurochemistry and Biological Markers Unit of the 1st Department of Neurology, “Eginition” Hospital) assessed CSF samples from 135 patients, using double sandwich enzyme-linked immunosorbent assay (ELISA) in an automated Euroimmun Analyzer I (Euroimmun, Lübeck, Germany), performed with commercially available kits (EUROIMMUN Beta-Amyloid (1–42) ELISA, EUROIMMUN Beta-Amyloid (1–40) ELISA, EUROIMMUN pTau(181) ELISA and EUROIMMUN Total-Tau ELISA, respectively; Euroimmun, Lübeck, Germany), as described elsewhere [21]. The cut-off levels of abnormality for this laboratory were Aβ_42_ ≤ 690 pg/mL, Aβ_42_/Aβ_40_ ≤ 0.105, τ_P-181_ *≥* 60 pg/mL, τ_T_ ≥ 400 pg/mL and τ_P-181_/Aβ_42_ ≥ 0.09.

Laboratory 2 (Department of Clinical Biochemistry of “Attikon” Hospital) assessed CSF samples from 107 patients using double sandwich ELISA performed with commercially available kits (“Innotest^®^ hTau antigen”, “β- amyloid1–42”, “β- amyloid1–40”and “phospho-tau181”, respectively; Fujirebio, Gent, Belgium), according to the manufacturer’s instructions. The cut-off levels of abnormality for this laboratory were Aβ_42_ ≤ 520 pg/mL, Aβ_42_/Aβ_40_ ≤ 0.044, τ_P-181_ ≥ 52 pg/mL, τ_T_ ≥ 375 pg/mL and τ_P-181_/Aβ_42_ ≥ 0.068. In the event of conflicting/marginal results, the CSF was cross-tested by the new CE-IVD Roche reagents that employ the Cobas 8000 automated platform in the e801 immunochemical analyzer, with a cut-off value for abnormality of a τ_P-181_/Aβ_42_ ratio > 0.023.

Laboratory 3 (Tzartos NeuroDiagnostics) assessed CSF samples from 32 patients using chemi-luminescence measured with a Lumipulse 600G automatic analyzer (Fujirebio, Gent, Belgium), as reported previously [54]. The cut-off values of abnormality for this laboratory were Aβ42 ≤ 520 pg/mL, Aβ42/Aβ40 ≤ 0.063, τ_P-181_ ≥ 60 pg/mL, τ_T_ ≥ 360 pg/mL and τ_P-181_/Aβ_42_ ≥ 0.09.

For external quality control, laboratories 1 and 3 participated in “The Alzheimer’s Association’s QC program for CSF and blood biomarkers” [55]. Laboratory 2 followed the External Quality Assessment (EQA) for both CSF biomarkers and *APOE* genotyping by INSTAND e.V., an ISO17043-accredited organization [56]

### 4.4. CSF Biomarker Profiling

Following assessments of CSF biomarkers, the profile of each patient was determined according to the AT(N) classification system [5]. The AD profile was a priori defined as A^+^T^+^ [5,9,17]. Since some patients with AD may present with normal or marginal τ_P-181_ levels [18,19,20,21], patients with the A^+^T^−^ profile (as well as A^−^T^+^ and A^−^T^−^ profiles) were further tested for possible evidence of AD. Since amyloid PET studies are costly and are not reimbursed in Greece, the hybrid τ_P-181_/Aβ_42_ ratio, which may helpful in doubtful cases [22], was used instead [6]. All of the A^−^T^+^ and A^−^T^−^ patients showed normal τ_P-181_/Aβ_42_, suggesting a non-AD diagnosis. Some of the A^+^T^−^ patients showed increased τ_P-181_/Aβ_42_, which was considered suggestive of AD presence. When the A^+^T^−^ profile was accompanied by normal τ_P-181_/Aβ_42_, this was considered to be an indication of amyloid pathology in the absence of concomitant tangle pathology, placing the patient somewhere on the Alzheimer’s *continuum*, but not indicating the presence of *Alzheimer’s disease* [5].

### 4.5. APOE Genotyping

Genotyping of *APOE* was performed at the Department of Clinical Biochemistry of “Attikon” Hospital as previously described [11]. In brief, blood was collected in EDTA-containing tubes and centrifuged. Genomic DNA was extracted using the “High Pure PCR Template Kit” (Roche, Mannheim, Germany) and amplified using a “real-time qPCR kit” (TIB MolBiol, Berlin, Germany) with the “Light Cycler PCR” platform (Roche, Mannheim, Germany).

### 4.6. Statistical Analysis

All numerical variables were checked for normality and homogeneity of variances by Shapiro–Wilk and Leven’s tests, respectively, and parametric or nonparametric tests were used appropriately. For CSF τ_P-181_, τ_T_ and τ_P-181_/Aβ_42_, deviations from normality and/or homogeneity of variances were noted. Logarithmic transformation restored the above violations and permitted the use of ANOVA models. For dichotomous or categorical variables, χ^2^ or Fisher’s exact tests were used. Logistic regression models were also used for predicting AD biomarker profiles. For statistical analysis, the following software packages were used: Statistica version 8.0, 2008 (StatSoft Inc., Tulsa, OK, USA); Prism version 6.01, 2012 (GraphPad Software Inc., San Diego, CA, USA); and MedCalc ^®^ version 12.5, 2013 (MedCalc Software, Ostend, Belgium). The level of statistical significance was set at *p* < 0.05.

## Figures and Tables

**Table 1:** Demographic, clinical and biochemical data of the studied population according to the various *APOE* genotypes.

	ε4/ε4	ε4/ε3	ε4/ε2	ε3/ε3	ε3/ε2	ε2/ε2	*p* Value
*n* (%)	14 (5.1%)	72 (26.3%)	5 (1.8%)	161 (58.8%)	21 (7.7%)	1 (0.3%)	
Gender (M/F)	5/9	26/46	1/4	67/94	8/13	1/0	NS *
Age (y)	68.6 ± 5.2	68.9 ± 8.4	66.0 ± 14.4	72.1 ± 8.9 ^a^	69.9 ± 7.4	83.0	0.044 ^†^
Duration (y)	3.2 ± 2.0	3.7 ± 3.7	5.5 ± 0.7	3.6 ± 3.4	3.9 ± 3.6	1.5	NS ^†^
Onset (y)	65.4 ± 6.1	65.2 ± 8.3	60.4 ± 13.4	69.5 ± 8.3 ^b^	66.1. ± 8.7	81.5	0.0064 ^†^
Education (y)	11.0 ± 5.9	12.0 ± 4.3	7.5 ± 2.1	10.6 ± 5.0	9.1 ± 4.0	6.0	NS ^†^
MMSE score	19.6 ± 5.8 21 (14–23)	21.6 ± 7.3 24 (17–27)	26.5 ± 3.5 27 (24–29)	22.8 ± 5.8 24 (21–27)	22.6 ± 5.4 24 (18–26)	25	NS ^†^ NS ^‡^
ACE-R total score	57.9 ± 18.4	65.0 ± 20.7	82.5 ± 3.5	66.1 ± 18.6	63.4 ± 20.8	65	NS ^†^
Attention-orientation	13.0 ± 3.9	13.7 ± 4.7	16.1 ± 1.2	14.2 ± 3.9	14.6 ± 3.7	17	NS ^†^
Memory	10.4 ± 4.9	14.2 ± 6.9	19.0 ± 4.2	14.8 ± 6.6	15.0 ± 7.0	19	NS ^†^
Fluency	5.2 ± 3.5	5.9 ± 3.6	9.0 ±1.4	5.5 ± 3.3	5.8 ± 3.3	3	NS ^†^
Language	17.7 ± 6.3	20.7 ± 5.0	24.0 ± 1.4	20.2 ± 4.9	17.5 ± 6.3	14	NS ^†^
Visuospatial	11.7 ± 2.8	10.7 ± 3.9	13.5 ± 2.1	11.6 ± 3.1	9.9 ± 4.2	12	NS ^†^
MTA score	3 (1.5–3)	2.5 (1–3)	0.5 (0–3)	2 (1–3)	2 (0.1–2.9)	3	NS ^‡^
ERICA score	2 (1.5–2)	2 (0.5–2)	1.5 (0–2)	1 (1–2) ^c^	1 (0–1) ^c^	2	0.0016 ^‡^
Aβ_42_ (pg/mL)	457 ± 143	564 ± 249	767 ± 384	710 ± 379	788 ± 461	626	0.003 ^#^
Aβ_40_ (pg/mL)	7471 ± 2235	10077 ± 5482	9988 ± 2655	9171 ± 5238	9347 ± 3433	6852	NS ^#^
Aβ_42_/Aβ_40_	0.069 ± 0.019	0.069 ± 0.032	0.076 ± 0.032	0.093 ± 0.047	0.114 ± 0.050	0.091	<0.001 ^#^
τ_P181_ (pg/mL)	94.7 ± 47.3 86 (55–118)	83.9 ± 50.9 72 (46–114)	45.4 ± 20.1 39 (33–58)	61.5 ± 41.1 49 (32–81)	52.9 ± 23.2 48 (38–62)	35	NS ^#^
τ_T_ (pg/mL)	520 ± 247 500 (361–626)	512 ± 391 419 (257–588)	269 ± 83.2 246 (218–320)	384 ± 285 298 (178–487)	460 ± 269 357 (268–718)	376	NS ^#^
τ_P181_/A_β42_	0.21 ± 0.13 0.16 (0.12–0.26)	0.18 ± 0.13 0.16 (0.08–0.24)	0.07 ± 0.04 0.07 (0.04–0.10)	0.13 ± 0.11 0.10 (0.05–0.17)	0.12 ± 0.17 0.06 (0.05–0.10)	0.06	NS ^#^

Data for all *APOE* genotypes are presented as the mean ± standard deviation or the median value (25th–75th percentile). Statistical comparisons were performed between ε4 homozygotes (ε4/ε4), ε4 heterozygotes (combined ε4/ε3 and ε4/ε2 groups), the ε3/ε3 group and the combined ε3/ε2 and ε2/ε2 groups. * χ^2^ test; ^†^ One-way ANOVA followed by Holm–Sidak’s test for multiple comparisons; ^‡^ Kruskal–Wallis test followed by Conover’s post hoc comparisons; ^a^ *p* = 0.025 vs. ε4 heterozygotes; ^b^ *p* = 0.0025 vs. ε4 heterozygotes; ^c^ *p* = 0.05 vs. e4 homozygotes and 0.05 vs. ε4 heterozygotes. ^#^ Analysis of covariance with age as a covariate and disease type (AD vs. non-AD), ε4 presence or absence, laboratory and sex as cofactors.

**Table 2:** Observed CSF biomarker profiles and percentages for the various *APOE* genotypes.

	ε4/ε4	ε4/ε3	ε4/ε2	ε3/ε3	ε3/ε2	ε2/ε2
*n* (%)	14 (5.1%)	72 (26.3%)	5 (1.8%)	161 (58.8%)	21 (7.7%)	1 (0.3%)
*Total suggestive of AD pathology*	14 (100%)	52 (72.2%)	1 (20%)	63 (39.1%)	6 (28.6%)	0 (0%)
A^+^T^+^	12 (85.7%)	46 (63.9%)	1 (20%)	53 (32.9%)	5 (23.8%)	0 (0%)
A^+^T^−^ with abnormal τ_P181_/A_β42_ ratio	2 (14.3%)	6 (8.3%)	0 (0%)	10 (6.2%)	1 (4.8%)	0 (0%)
*Total suggestive of non-AD pathology*	0 (0%)	20 (27.8%)	4 (80%)	98 (60.9%)	15 (71.4%)	1 (100%)
A^+^T^−^ with normal τ_P181_/A_β42_ ratio	0 (0%)	6 (8.3%)	1 (20%)	21 (13.1%)	2 (9.5%)	0 (0%)
A^−^T^+^	0(0%)	1 (1.4%)	1 (20%)	16 (9.9%)	3 (14.3%)	1 (100%)
A^−^T^−^	0 (0%)	13 (18.1%)	2 (40%)	61 (37.9%)	10 (47.6%)	0 (0%)

**Table 3:** Comparison of *APOE* levels with ε4 presence vs. absence in AD CSF biomarker profiles vs. all other (non-AD) profiles.

	*p* Value ^a^	Odds ratio ^b^	Sensitivity ^b^	Specificity ^b^	PPV ^b^	NPV ^b^
At least one ε4 allele ^1^	<0.0001	5.87 (3.35–10.3)	0.53 (0.44–0.62)	0.84 (0.77–0.89)	0.74 (0.63–0.82)	0.68 (0.60–0.74)
ε4/ε3 and ε4/ε2 ^1^	<0.0001	4.61 (2.62–8.24)	0.47 (0.38–0.57)	0.84 (0.77–0.89)	0.69 (0.52–0.79)	0.68 (0.60–0.74)
ε4/ε4 ^1^	<0.0001	60.7 (3.56–1035)	0.19 (0.11–0.30)	1.00 (0.97–1.00)	1.00 (0.77–1.00)	0.68 (0.60–0.74)
ε4/ε4 ^2^	<0.0001	38.3 (2.26–649)	0.11 (0.06–0.18)	1.00 (0.98–1.00)	1.00 (0.77–1.00)	0.57 (0.51–0.63)

^a^ Fisher’s exact test. ^b^ Values in parentheses indicate 95% confidence interval. PPV: positive predictive value. NPV: negative predictive value. ^1^ vs. absence of ε4. ^2^ vs. absence of ε4 or ε4 heterozygosity. Values in parentheses indicate 95% confidence intervals.

**Table 4:** Logistic regression models taking into account the possible effect of sex and education.

	*p* Value	Odds Ratio	AUC
*First model*: ε4 presence vs. ε4 absence			
Model	<0.0001		0.75 (0.68–0.81)
Education	NS	0.97 (0.90–1.04)	
Sex	0.03	2.04 (1.20–3.45)	
At least one ε4 allele	<0.0001	6.31 (3.12–12.76)	
*Second model*: others vs. ε3/ε3 as reference			
Model	<0.0001		0.76 (0.69–0.82)
Education	NS	0.96 ((0.89–1.03)	
Sex	0.04	1.9 (1.03–3.88)	
ε4/ε3 and ε4/ε2	0.0001	4.46 (2.15–9.25)	
ε4/ε4	0.05	37.7 (2.1–501)	
ε3/ε2 and ε2/ε2	NS	0.37 (0.09–1.03)	

## Data Availability

The data presented in this study are available upon request from the corresponding author. The data are not publicly available due to privacy restrictions.

## Abbreviations

The following abbreviations are used in this manuscript:

AD	Alzheimer’s disease
APOE	Apolipoprotein E
MMSE	Mini Mental State Examination
ACE-R	Addenbrooke’s Cognitive Examination—Revised version
MTA	Medial Temporal Atrophy score
ERICA	Entorhinal Cortex Atrophy score
ANOVA	Analysis of Variance
CBS	Corticobasal syndrome
FTD	Frontotemporal dementia
PSP	Progressive supranuclear palsy
PCA	Posterior cortical atrophy
DLB	Dementia with Lewy bodies
VCI	Vascular cognitive impairment
CAA	Cerebral amyloid angiopathy
NPH	Normal-pressure hydrocephalus
